# Hydrolytic Enzymes in the Secretome of the Mushrooms *P. eryngii* and *P. ostreatus*: A Comparison Between the Two Species

**DOI:** 10.3390/molecules30122505

**Published:** 2025-06-07

**Authors:** Tania Petraglia, Tiziana Latronico, Grazia Maria Liuzzi, Angela Fanigliulo, Aniello Crescenzi, Rocco Rossano

**Affiliations:** 1 Department of Basic and Applied Sciences, University of Basilicata, 85100 Potenza, Italy; tania.petraglia@unibas.it; 2 Department of Biosciences, Biotechnologies and Environment, University of Bari “Aldo Moro”, 70126 Bari, Italy; tiziana.latronico@uniba.it; 3 Bioagritest Srl-Centro Interregionale di Diagnosi Vegetale, 85010 Pignola, Italy; bioagritest@gmail.com; 4 Department of Agricultural, Forestry, Food and Environmental Sciences, University of Basilicata, 85100 Potenza, Italy; aniello.crescenzi@unibas.it

**Keywords:** mushroom substrate, hydrolytic enzymes, secretome, mushroom cultivation, cellulases, hemicellulases, proteases, lipases

## Abstract

The fungi belonging to the genus *Pleurotus* can be cultivated in different substrates and represent excellent producers of several extracellular enzymes. In this study, we analyzed eleven hydrolytic enzymes of the *P. eryngii* and *P. ostreatus* secretomes, which were collected at three different growth stages after 23 days (mycelial colonization of about 50% of the substrate), 34 days (100% colonization of the substrate) and 50 days (after the first flush). Mushrooms were axenically cultivated on the same substrate. The results demonstrate that proteases, lipases, amylases, α-glucosidase, cellulases (endoglucanase, β-cellobiohydrolase and β-glucosidase) and hemicellulase (xylosidase, glucuronidase, arabinosidase and mannosidase) activities were higher in the secretomes from *P. eryngii* than those from *P. ostreatus*. Time course analysis revealed for both species a similar enzymatic activity profile, in which in the early stages of mycelium development, both species use starch as the main carbon source. Protease and lipase activities increased and remained constant during the subsequent formation of fruiting bodies, whereas cellulase and hemicellulase activities decreased after the complete mycelial colonization of the substrate. The zymographic analysis suggested the presence in the secretomes of proteolytic activities belonging to different classes. In conclusion, both mushroom species released into the secretomes a broad spectrum of hydrolytic enzymes potentially useful in various biotechnological fields.

## 1. Introduction

White-rot fungi are able to use different types of agroindustrial waste for their growth and are able to secrete several enzymes for degrading polymers such as cellulose, hemicellulose, lignin and proteins into low-molecular-weight compounds which are assimilated for their growth. To this regard, many studies are focused on the recovery and utilization of these extracellular enzymes of great commercial interest that residue in the spent mushroom substrate (SMS) post-harvest [1,2,3,4,5]. The main group is represented by lignocellulosic enzymes followed by proteases [6,7]. The decomposition of lignocellulosic materials is achieved by the synergic action of hydrolytic enzymes for cellulose and hemicellulose deconstructing and oxidative enzymes involved in lignin degradation [7,8]. The hydrolysis of cellulose requires three types of hydrolytic enzymes: endoglucanases (EC 3.2.1.4), cellobiohydrolases (EC 3.2.1.91) and β-1,4-glucosidases (EC 3.2.1.21). Regarding the degradation of hemicellulose, the main enzymes are xylanase (EC 3.2.1.8), β-xilosidase (EC 3.2.1.37), α-glucuronidase (EC 3.2.1.139), β-mannosidase (EC 3.2.1.25) and α-arabinofuranosidase (EC 3.2.1.55) [9]. Lignin degradation is due to the combined action of two groups of enzymes, such as phenol oxidase (laccase, EC 1.10.3.2) and peroxidases (lignin peroxidase, EC 1.11.1.14; manganese peroxidase, EC 1.11.1.13; versatile peroxidase; and dye-decolorizing peroxidase, EC 1.11.1.19) [7].

In this regard, the use of species belonging to the *Pleurotus* genus could be very advantageous when compared with other mushrooms, since, as well as being excellent enzyme producers, they can be easily grown on different substrates, require low amounts of nutrient and show excellent adaptation. *Pleurotus* species are commercially cultivated on various raw materials containing high lignocellulosic substances such as sawdust, straw, hay, cottonseed hulls, and corn cobs. Their efficacy in using nutrients from lignocellulose residues is due to a set of potent lignocellulolytic enzymes that consist of cellulases, hemicellulases, laccases and peroxidases, which degrade different polysaccharidic and aromatic compounds [10,11,12,13,14]. As previously reported, after lignocellulosic enzymes, proteases represent the second largest group of hydrolytic enzymes secreted by fungi into the growth substrate. Proteases play important roles in the physiology of fungi; they are essential for absorbing nitrogen from the environment and in processes such as germination and sporulation [15]. Also, as reported in the *Pleurotus* genus, proteases seem to be involved in the regulation of other extracellular enzymes at certain stages of fungal growth [16,17,18,19]. There is growing commercial interest for this group of enzymes due to their numerous industrial applications as pharmaceutical agents, detergents, leather, and food [15]. Proteases account for more than 60% of the total worldwide enzyme [20]. Currently, ninety percent of marketed proteases are derived from microorganisms; only recently research for new proteases with specific characteristics in *Basidiomycetes* and, in particular, in *Pleurotus* spp. acquired a certain importance [21,22]. Another group of hydrolases secreted by fungi during their growth is the lipases which represent the third biggest family of hydrolytic enzymes after carbohydrate-processing enzymes and proteases. Fungal lipases are enzymes that have applications in numerous biotechnological fields and sectors such as the production of bioethanol, paper and cosmetics; in food processing; in the textile industry; in the medical and pharmaceutical sector; in bioremediation; and in various biosynthetic processes [23]. However, for the lipolytic enzymes of *Pleurotus* fungi, unlike lignocellulosic enzymes, few studies are available in the literature [24,25,26,27,28].

Recently, approaches such as mass spectrometry-based proteomics have been widely employed in fungal secretome analysis to identify and quantify individual proteins [6,29].

In this study, classical biochemical methods have been employed in order to emphasize the functional activity of extracellular enzymes secreted under cultivation-controlled conditions. In particular we compared the main hydrolytic enzymes of secretomes recovered at three different growth stages from *P. eryngii* and *P. ostreatus* axenically cultivated on the same growth substrate. Results demonstrated that both species release into the secretomes a broad spectrum of hydrolytic enzymes, such as proteases, lipases, cellulases and hemicellulases, which are potentially useful in various biotechnological fields.

## 2. Results and Discussion

### 2.1. Substrate Collection

**Figure 1:**
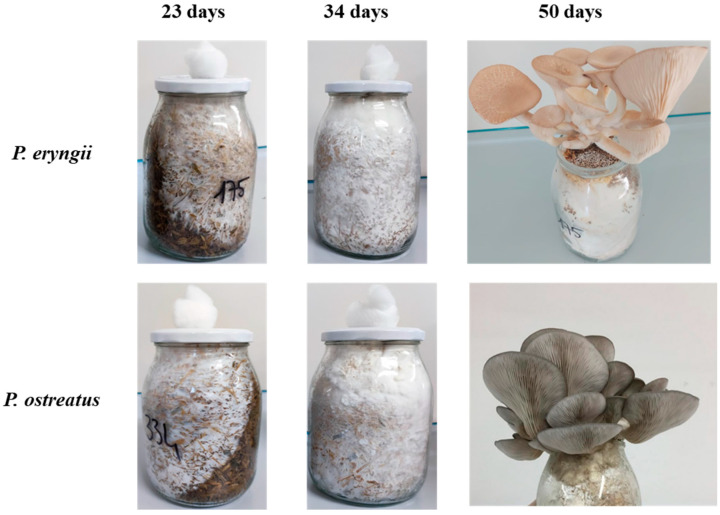
The mushroom substrate derived from the axenically cultivated *P. eryngii* and *P. ostreatus* used for the preparation of secretomes. Substrates were collected at three growth stages: 23, 34 and 50 days.

Secretomes were collected by liquid extraction from the substrates colonized by the mycelia at three different stages (Figure 1). Enzymes were recovered after 23 days of incubation, that is, when the mycelia had colonized approximately 50% of the substrate; after 34 days, corresponding to the complete substrate colonization by the mycelia; and at 50 days, which is immediately after mushroom harvest.

### 2.2. Protein Content

As reported in Table 1, during the development of the mycelium, between 23 and 34 days of incubation, for both *Pleurotus* species, the protein content per gram of moist substrate increased, whereas it decreased with the development of fruiting bodies. During the development of the mycelium, the highest quantity of proteins was measured in the secretome from both *Pleurotus* species collected after 34 days (2.54 ± 0.16 mg prot/g for *P. eryngii* and 1.79 ± 0.14 mg prot/g for *P. ostreatus*, respectively), which was approximately double that determined after 23 days (1.33 ± 0.11 mg prot/g for *P. eryngii* and 0.94 ± 0.06 mg prot/g for *P. ostreatus*, respectively).

Secretomes collected at 50 days, that is, after the harvesting of fruiting bodies, showed protein content (1.97 ± 0.15 for *P. eryngii* and 1.26 ± 0.11 mg prot/g for *P. ostreatus*) approximately 72% and 70% than the highest level, respectively. Regarding the comparison among the two species, for all the samples, secretomes of *P. eryngii* showed higher protein concentrations than *P. ostreatus.*

These results are in agreement with what has been reported by Xie et al., 2016 [29], on the secretome of *P. eryngii* cultivated on the ramie stalk substrate, in which the protein content from the primordial initiation stage of incubation was greater than that of other growth stages.

### 2.3. Total Proteolytic Activity Assessment and Zymographic Analysis

**Figure 2:**
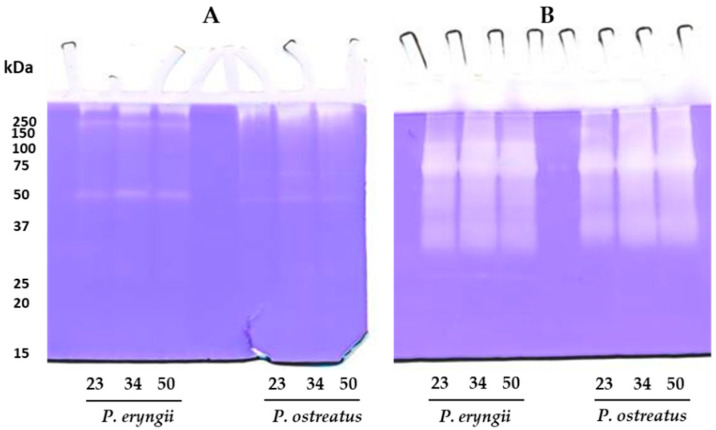
Analysis of caseinolytic (gel (**A**)) and gelatinolytic (gel (**B**)) activities by zymography. Zymograms (10% polyacrylamide/0.1% casein or gelatin) of the secretomes after 23, 34 and 50 days of incubation. For each sample, 1 μg of protein was applied onto the gels.

Total proteolytic activity of secretomes collected at different stages of the mycelial colonization of the substrate and immediately after mushroom harvest was assessed spectrophotometrically using azocasein as the substrate. Results for both species show that during the development of the mycelia, the production of proteases increased and remained constant during the subsequent formation of fruiting bodies (Table 1). *P. eryngii* exhibited the maximum total proteolytic activity (773.93 ± 9.34 U/g), about 1.3 times higher than that found for *P. ostreatus* (594.95 ± 13.06 U/g). As already reported, the amount of proteolytic enzymes secreted by fungi into the growth medium during their development is strictly dependent on the environment in which they are grown in, as well as the type of protein substrate on which they act [6]. The fact that, in the secretomes collected after 50 days, the levels of proteases remain high could suggest that while in the developmental phases, they contribute to nitrogen recovery; subsequently, they could be involved in the maintenance of protein homeostasis through the regulation of other enzymatic systems. In fact, as reported in the *Pleurotus* genus, at certain stages of fungal growth, proteases seem to be involved in the regulation of other extracellular enzymes such as laccases and peroxidases [16,17,18,19,30]. To determine the composition and the molecular mass of the proteases present in the secretomes collected from the growth substrate, protease activity was analyzed by mono-dimensional zymography. As shown in Figure 2, the zymographic analysis highlighted a different proteolytic pattern when secretomes were analyzed on gels copolymerized with casein (Figure 2A) or gelatin (Figure 2B).

**Figure 3:**
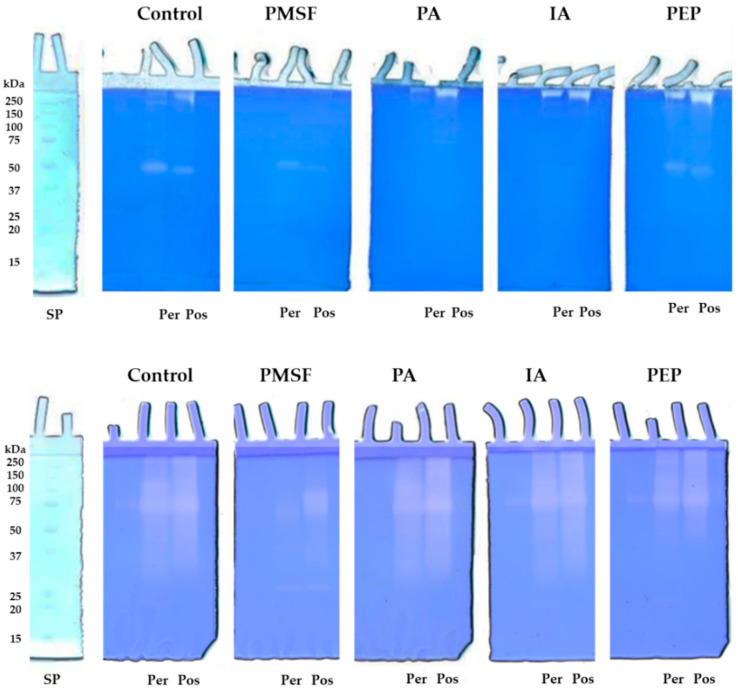
Analysis of caseinolytic (**upper panel**) and gelatinolytic (**lower panel**) activity by zymography. Zymograms (10% polyacrylamide/0.1% casein or gelatin) of secretomes after 34 days of incubation. Samples were analyzed in the absence (control) and in the presence of specific inhibitors: phenylmethylsulfonyl fluoride (PMSF), 1,10 phenanthroline (PA), iodoacetamide (IA) and pepstatin (PEP). Per: *Pleurotus eryngii*; Pos: *Pleurotus ostreatus*.

In particular, in the casein zymogram (Figure 2A), both secretomes showed a proteolytic pattern characterized by the presence of a major well-defined digestion band of about 50 kDa for *P. eryngii* and 47 kDa for *P. ostreatus*. Furthermore, in the upper region of the gel, in the case of *P. eryngii*, two additional high-molecular-weight bands were evidenced, whereas, in the *P. ostreatus* secretome, only one band was observed. Figure 2B shows the electrophoretic profile of gelatinolytic enzymes. The analysis revealed for both secretomes the presence of a not well-resolved proteolytic pattern. However, for *P. eryngii*, two bands of approximately 120 and 70 kDa can be distinguished, whereas for *P. ostreatus*, only this latter was seen. As evidenced from the analysis of the total proteolytic activity of secretomes collected at different stages of growth, the production of proteases increased at 34 days and remained constant during the subsequent formation of fruiting bodies (50 days), both on casein and gelatin zymography. To characterize the nature of these proteases, an analysis was performed in the presence of different protease inhibitors. The zymographic analysis on casein-copolymerized gels (Figure 3, upper panel) in the presence of PMSF, a specific inhibitor for serine proteinases, showed, for both the *Pleurotus* species, a decrease (about 55%) in their major proteolytic bands in comparison to the control in the absence of inhibitors (Control). As for the high-molecular-weight bands, they disappeared completely in the presence of PMSF, suggesting that these enzymatic activities could not be identified as aggregates or complexed forms of the bands of 50 and 47 kDa but rather correspond to serine proteases. On the contrary, in gels corresponding to the samples incubated with 1,10 phenanthroline (PA) (metalloproteinases inhibitor) and iodoacetamide (IA) (a specific inhibitor for cysteine proteases), the high-molecular-weight bands were not inhibited, whereas the major band was completely inhibited, suggesting that this latter band belongs to the class of metalloproteases that are probably thiol-dependent. The different behaviours of the high-molecular-weight bands towards PA and IA support the hypothesis that these bands do not correspond to multimeric forms of the major proteolytic bands. Finally, pepstatin (PEP) (a specific inhibitor for aspartic proteases) did not affect any proteolytic activity present on casein-copolymerized gels.

Regarding the effect of the inhibitors on the proteolytic activities present on gelatin zymography (Figure 3, lower panel), a reduction in all the proteolytic activities present in the control was observed only in the samples incubated in the presence of PMSF, suggesting that these activities could be ascribed to serine proteases. In our previous study [31] conducted on crude extracts from the same strains of *P. eryngii* and *P. ostreatus*, we demonstrated the presence of different fibrinolytic enzymes belonging to serine proteases and metalloproteases. In this regard, as reported by Inácio et al. [15], most proteases of the genus *Pleurotus* feature the characteristics of alkaline subtilases, a group of serine proteases, whereas aspartic proteases are rarer. In their study, Sabotic et al. [32] carried out gelatin zymography on aqueous extracts of 43 basidiomycetes species and showed a predominance of serine proteases.

### 2.4. Carbohydrate-Hydrolyzing Enzymes

**Figure 4:**
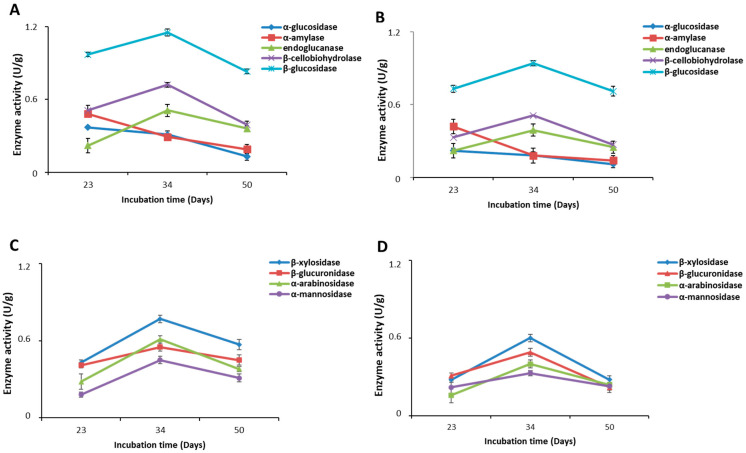
Enzymatic activity of the *P. eryngii* (**A**,**C**) and *P. ostreatus* (**B**,**D**) secretomes recovered from the substrate at three growth stages: after 23, 34 and 50 days of incubations. The enzymatic activities were evaluated spectrophotometrically as described in the Materials and Methods Section. Values of cellulolytic, amylolytic (**A**,**B**) and hemicellulolytic activity (**C**,**D**) are reported as the mean ± SD of two extracts analyzed in triplicate (n = 6). Statistically significant differences (one-way ANOVA followed by Tukey’s post hoc test, *p* < 0.05) are reported in Table 2.

Carbohydrate-hydrolyzing enzymes are involved in the synthesis, metabolism and transport of carbohydrates. Figure 4A,B show the time course of the amidolytic and cellulolytic activities in the secretomes of *P*. *eryngii* and *P. ostreatus,* respectively. Regarding α-amylase and α-glucosidase, both enzymes showed decreasing values over time. In particular, as reported in Table 2, the highest activity values were observed in the sample recovered after 23 days, while after 34 and 50 days, the enzyme’s activity showed a statistically significant decrease (*p* < 0.05) over time. In contrast, the three cellulolytic enzymes, namely endoglucanase, β-cellobiohydrolase and β-glucosidase, presented a statistically significant peak of activity after 34 days in correspondence with the complete colonization of substrate by the mycelium, with a statistically significant decrease (*p* < 0.05) after 50 days. In the case of *P. ostreatus* (Figure 4B), for all enzymes, lower activity values were measured compared to those of *P. eryngii*. The trend of both α-amylase and α-glucosidase over time suggests that both *Pleurotus* species during the early stages of development of mycelium predominantly use starch as a carbon source, while during the complete colonization of the substrate and the subsequent formation of the fruiting bodies, they mainly degrade the cellulosic material.

Together with cellulase activities, hemicellulases represent another main type of carbohydrate-hydrolyzing enzyme in white-rot secretomes [33]. In this work, four enzymes involved in the degradation of hemicellulose were analyzed: xylosidase, glucuronidase, arabinosidase and mannosidase. Similarly to what was previously described for cellulolytic enzymes, the time course of the hemicellulases (Figure 4C,D) secreted into the growth substrate by both mushrooms was characterized by a statistically significant increase (*p* < 0.05) in their activity in the period between 23 and 34 days of incubation followed by a statistically significant decrease (*p* < 0.05) during the formation of the fruiting bodies. *P.eryngii* (Figure 4C) presented higher activity values than *P. ostratus* (Figure 4D) for all the studied enzymes. The most active enzyme for both mushrooms was xylosidase (Table 2), a member of the xylanase enzymes that breakdown xylan into xylose. Xie et al. [29] analyzed the secretome of *P. eryngii* grown on a substrate different from that used in this study and reported the profile over time of six different enzymatic activities, some of which were analyzed also in this study. Although the substrate used by these authors resulted in slower growth for the mushroom, considering the stage of development of the mushroom, it is possible to observe, for the different enzymatic activities considered, a time course in line with that observed in this study.

### 2.5. Lipolytic Activity

**Figure 5:**
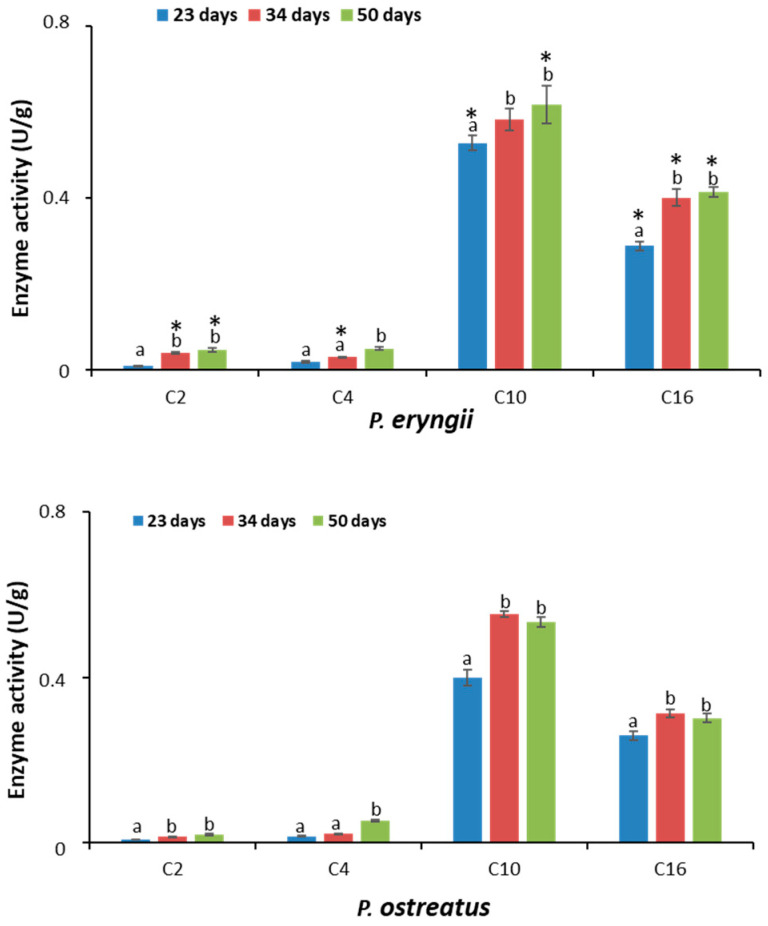
Lipolityc activity of secretomes recovered at three growth stages: after 23, 34 and 50 days of incubations. Lipolytic activity was evaluated spectrophotometrically using p-nitrophenyl labelled substrates: p-nitrophenyl acetate (C2), p-nitrophenyl butyrate (C4), p-nitrophenyl decanoate (C10) and p-nitrophenyl palmitate (C16). Values are reported as the mean ± SD of two extracts analyzed in triplicate (n = 6). For each species, different letters indicate a statistically significant difference. Asterisks indicate a statistically significant difference between the two species at each time point (one-way ANOVA followed by Tukey’s post hoc test, *p* < 0.05).

The lipolytic activity was evaluated spectrophotometrically using pNP-labelled substrates with different acyl chain lengths (Figure 5).

For both species, the lipases present in the secretomes showed greater activity towards long-chain substrates. It would seem that the lipases of both *P. eryngii* and *P. ostratus* preferentially hydrolyzed acyl chains with the following order: C10 > C16 > C4 ≥ C2. Regarding the time course analysis of extracellular lipase production, the secretome profile of the two species was comparable. As shown in Figure 5, except for the C4 substrate, the highest activity values were observed in the samples collected after 34 and 50 days of incubation.

The comparison between the two species showed that in the secretome collected after 23 days, *P. eryngii* presented greater activity compared to *P. ostreatus* (*p* < 0.05) for both p-nitrophenyl decanoate (C10) and p-nitrophenyl palmitate (C16) (0.53 ± 0.02 U/g vs. 0.40 ± 0.02 U/g and 0.29 ± 0.01 U/g vs. 0.24 ± 0.01 U/g, respectively). No statistically significant differences were observed towards the two short-chain substrates, namely p-nitrophenyl butyrate (C4) and p-nitrophenyl acetate (C2) (0.020 ± 0.006 U/g vs. 0.016 ± 0.002 U/g and 0.010 ± 0.001 U/g vs. 0.008 ± 0.001 U/g, respectively). By contrast, after 34 days, with the exception of the C10 substrate, whereby no differences were observed, lipases secreted by *P. eryngii* were more active than those of *P. ostreatus* (*p* < 0.05). Finally, after 50 days, with the exception of the C4 substrate, whereby no differences were observed, *P. eryngii* was more active than *P. ostreatus* (*p* < 0.05) on all the other substrates. Data are summarized in Table 3.

Based on the preference towards fatty acid chain lengths, the enzymatic activities of both secretomes could be classified as true lipases [34]. In this regard, in the study conducted by Piscitelli et al. [26] on the extracellular lipases produced from *P. ostreatus* in the presence of different carbon sources (olive oil, OMW, glycerol and glucose), two of the four identified lipases (PleoLip241 and PleoLip369) preferentially hydrolyzed long-chain substrates with preference towards C10. It can be hypothesized that the presence of lipolytic activity in the secretomes analyzed in this study could be related to the presence of the growth substrate of corn chips, which act as lipase inducers. In support of this observation, Dedousi et al. [27] reported that for *Pleurotus eryngii* and *ostreatus* species, the use of growth substrates integrated with oil makes them powerful lipase producers. In particular, for *P. ostreatus*, the highest extracellular lipase activities was detected when it was cultivated on wheat straw supplemented with 5% *w*/*w* sunflower oil, whereas in the case of *P. eryngii,* the highest lipase activity was achieved in the presence of 2% *w*/*w* corn oil.

## 3. Materials and Methods

### 3.1. Mushroom Cultivation and Substrate Collection

*P. eryngii* (Bio 175/Pe strain) and *P. ostreatus* (Bio 334/Po strain), which were stored at the Bioagritest (Interregional Center for Plant Diagnosis, Pignola, Italy) mushroom library, were used. The two strains were grown on acidified potato dextrose agar (PDA) containing the antibiotic streptomycin sulphate (100 m g/L) at a constant temperature of 24 °C and then sub-cultured and maintained on the same substrate at 5 °C for up to 5 months. The substrate (4.1% wheat straw, 4.1% corn chips, 4.1% thistle wood chips, 4.1% spelled chaff, 15% sugar beet, and 3.6% CaCO_3_; moisture was adjusted to 65% with water) was prepared in 1000 mL polypropylene bottles with aerated caps and sterilized in an autoclave at 121 °C for 90 min and then cooled to room temperature. Thereafter, 1 cm Ø PDA discs containing the mycelium were inoculated and incubated at 22–24 °C with 8 h of light (50 lux) and 16 h of darkness until the substrate was completely colonized. The colonization rate was measured every 5 days in both vertical and circular (depth) directions. When the entire volume of the substrate was colonized by fungal mycelium, bottles were transferred to a cold room at 10 °C in the dark for 5 days to induce fruiting body differentiation. After mycelium differentiation, bottles were opened and placed in a production cell at a temperature of 16 °C for 10 h and 22 °C for 14 h with a relative humidity of approximately 85% and a CO_2_ concentration not exceeding 800 ppm. In all tests, fruiting bodies were harvested when they reached the optimal size for commercial use (complete opening of the cap). Substrates from the axenically cultivated mushrooms were collected at three growth stages: after 23 days (when the mycelium colonized 50% the substrate), 34 days (when the mycelium completely colonized the substrates) and 50 days (after the first flush). Samples were placed in plastic bags and stored under vacuum conditions at −80 °C.

### 3.2. Extraction of Extracellular Enzymes

Extracellular enzymes were extracted in duplicate from the substrates by suspending 5 g of substrate in 75 mL of 40 mM sodium acetate buffer at pH 5.5. Samples were incubated with shaking at 170 rpm for 4 h on ice, filtered on Whatman 3 paper discs and centrifuged (12,000× *g* for 20 min at 4 °C). Supernatants representing the crude secretomes were assayed for enzymatic activities or lyophilized and stored at −80 °C. Enzyme activities are expressed as U/g of the substrate. All assays were performed in triplicate.

### 3.3. Protein Content and Total Proteolytic Activity

Protein content was determined according to the Bradford method [35], and it was expressed as mg of protein per g of substrate. Total proteolytic activity was assessed by using the spectrophotometric method based on azocasein [31]. Briefly, 0.1 mL of secretome was added to 0.4 mL of 1% (*w*/*v*) azocasein (Sigma-Aldrich, St. Louis, MO, USA) in 40 mM Tris-HCl buffer at pH 7.0; then, the mixtures were incubated at 37 °C. The reaction was stopped after 60 min by adding 0.5 mL of 10% trichloroacetic acid (TCA) and centrifuged for 4 min at 12,000 rpm (Amicon MC-13 microcentrifuge; Amicon, Beverly, MA, USA). An equal volume of 4 N NaOH was added to the supernatants, and the absorbance at 440 nm was recorded. The assay included an appropriate blank, in which TCA was added before the substrate. Total proteolytic activity was reported as U/g of the substrate, where U corresponds to the amount of enzyme producing 0.001 absorbance units per min at 440 nm under the assay conditions.

### 3.4. Zymographic Analysis

The proteolytic activities of secretomes collected from substrates were also determined by zymography. Aliquots of samples containing 1 μg of proteins were supplemented with 20 μL of electrophoresis non-reducing loading buffer: 4% (*w*/*v*) SDS, 12% (*w*/*v*) glycerol, 0.01% (*w*/*v*) bromophenol blue and 50 mM Tris–HCl (pH 6.8). Samples were then separated under non-reducing conditions in a 10% (*w*/*v*) polyacrylamide gel copolymerized with 0.1% (*w*/*v*) casein or gelatin [36,37]. Stacking gels contained 4% (*w*/*v*) polyacrylamide. Electrophoresis was performed at 4 °C for 80 min at a constant 150 V using a Bio-Rad Miniprotean apparatus (Bio-Rad Laboratories, Hercules, CA, USA). After electrophoresis, the standard protein lane was cut and stained for 2 min with Coomassie Brilliant Blue, while the remaining part of the gel was washed (2 × 20 min) in 2.5% (*w*/*v*) Triton X-100 and 20 mM sodium acetate buffer at pH 5.5 (wash buffer) to remove SDS and then incubated for 16 h at 37 °C in 20 mM sodium acetate buffer at pH 5.5 (developing buffer). At the end of incubation, gels were stained with Coomassie Brilliant Blue, washed with deionized water and, after destaining, scanned using an ImageMaster DTS scanner (Pharmacia Biotech, Uppsala, Sweden). Proteolytic activities were detected as clear digestion bands on the blue background and were quantified by computerized image analysis through 1D scanning densitometry using the Image Master 1D programme (Pharmacia Biotech, Uppsala, Sweden). Caseinolytic and gelatinolytic activities were expressed as optical density (OD) x mm^2^, representing the scanning area under the curves which takes into account both the brightness and width of the substrate degradation zone. To determine the nature of the enzymes, samples were incubated for 1 h at 25 °C in the presence of different enzyme inhibitors (Sigma-Aldrich, St. Louis, MO, USA), namely iodoacetamide (30 mM) for cysteine proteinases, pepstatin A (2 µM) for aspartic proteinases, phenylmethylsulfonyl fluoride (PMSF) (4 mM) for serine proteinases and 1,10-phenanthroline (PA) (20 mM) for metalloproteinases, and then subjected to zymographic analysis as described above. After electrophoresis, the lanes were cut and incubated individually in inhibitor-containing developing buffer. The effect of the inhibitors on the proteolytic enzymes was assessed by measuring residual activity with respect to the control samples incubated without inhibitors.

### 3.5. Polysaccharide-Hydrolizing Activity

#### 3.5.1. Cellulolytic and Amylolytic Activities

Endoglucanase activity was determined by measuring the amount of glucose released from carboxymethyl cellulose (Sigma-Aldrich St. Louis, MO, USA) [38]. For the measurement of the activity, 0.5 mL of the secretome was added to 0.5 mL of a 2% (*w*/*v*) carboxymethyl cellulose solution prepared in a 40 mM sodium acetate buffer (pH 5.5) and incubated (with shaking) for 30 min at 50 °C. After that, the mixture was centrifuged for 5 min at 12,000× *g*, and the supernatant was used for the assay. Glucose estimation was performed using the glucose oxidase (GOD) and peroxidase (POD) method [39]. Results were obtained from a calibration curve using glucose as the standard, and the activity was expressed as U/g, where one unit (U) is defined as the quantity of enzyme which releases 1 μmol of glucose in 1 min. β-glucosidase, β-cellobiohydrolase and α-glucosidase activities were determined by monitoring the release of p-nitrophenol from three synthetic substrates (p-nitrophenyl-β-D-glucopyranoside, p-nitrophenyl-β-D-cellobioside and p-nitrophenyl-α-D-glucopyranoside (Sigma-Aldrich St. Louis, MO, USA). For the assay, 0.05 mL of 10 mM substrates prepared in a 40 mM sodium acetate buffer at pH 5.5 was mixed with the same volume of the secretome; then, 0.1 mL of the acetate buffer was added. After 30 min of incubation at 30 °C, the enzymatic reaction was stopped by adding 0.8 mL of 200 mM Na_2_CO_3_. The absorbance was measured at a wavelength of 405 nm, and the results were extrapolated from a calibration curve generated using 4-nitrophenol (2–100 nmol) and expressed as U/g, where one unit (U) is defined as the quantity of enzyme which releases 1 μmol of p-nitrophenol in 1 min.

Amylase activity was measured using the 3,5-dinitrosalicylic acid (DNS) reagent as described by Miller [40]. Twenty microliters of the secretome were added to 180 μL of 1% starch in distilled water and incubated at 50 °C for 60 min, followed by the addition of 200 μL of DNS to stop the reaction. The solution was boiled for 10 min, placed on ice for 5 min and then added with 800 μL of water. The amount of reducing sugar was determined using glucose as the standard by measuring absorbance at 575 nm. Amylase activity (1 U) was defined as the quantity of enzyme required for the release of 1 μmol of glucose per min.

#### 3.5.2. Hemicellulolytic Activity

Hemicellulolytic activities of secretomes were assayed spectrophotometrically by using the following synthetic 4-nitrophenyl-labelled substrates: 4-nitrophenyl β-D-xylopyranoside, 4-nitrophenyl β-D-glucuronide, 4-nitrophenyl α-L-arabinopyranoside and 4-nitrophenyl α-D-mannopyranoside (Sigma-Aldrich, St. Louis, MO, USA). The assays were performed under the same conditions described in Section 3.5.1, measuring absorbance at 405 nm, which corresponds to the release of p-nitrophenol from the synthetic substrates.

### 3.6. Detection of Lipolytic Activity

Lipolytic activity was evaluated spectrophotometrically using the following pNP-labelled substrates: p-nitrophenyl acetate, p-nitrophenyl butyrate, p-nitrophenyl decanoate and p-nitrophenyl palmitate (Sigma-Aldrich, St. Louis, MO, USA). For the assay, the reaction mixture consisted of 0.05 mL of the secretome, 0.05 mL of the 10 mM substrate in acetonitrile and 0.9 mL of the 20 mM phosphate buffer at pH 6.8. After 40 min of incubation at 30 °C, the absorbance of p-nitrophenol released was measured at 405 nm. The results obtained from a calibration curve generated using 4-nitrophenol (2–100 nmol) were expressed as U/g, where one unit (U) is defined as the quantity of enzyme which releases 1 μmol of p-nitrophenol in 1 min.

## 4. Conclusions

This study provides a comprehensive comparative analysis of the hydrolytic enzymes released into the secretomes of *Pleurotus eryngii* and *Pleurotus ostreatus*, highlighting the enzymatic potential of these fungi when cultivated under axenic and standardized conditions. Our results clearly demonstrate that both species are prolific producers of extracellular hydrolytic enzymes, including proteases, lipases, cellulases and hemicellulases. However, *P. eryngii* consistently exhibited higher enzymatic activities across the evaluated parameters compared to *P. ostreatus*. The temporal analysis of enzyme secretion revealed a dynamic modulation of activity patterns: During the early stages of mycelial development, both species primarily relied on starch degradation. As colonization progressed, there was a notable shift towards the degradation of more recalcitrant lignocellulosic substrates, with peak cellulase and hemicellulase activities observed at full colonization. Interestingly, protease activities increased steadily and remained relatively constant during fruiting body formation, suggesting additional physiological roles beyond nutrient acquisition, possibly related to enzymatic regulation and protein turnover during morphogenesis. Zymographic analyses further confirmed the complexity of the proteolytic systems in both secretomes. Similarly, lipolytic profiles suggested preferential activity towards long-chain substrates, confirming the presence of true lipases in both species, an aspect of notable biotechnological relevance. Taken together, these findings emphasize the remarkable versatility of the hydrolytic enzymes released into secretomes by the *Pleurotus* species.

In conclusion, the valorization of the spent mushroom substrate, through the recovery of enzymatic activities prior to disposal, closes the circle of fungal cultivation, transforming a problematic waste into a concrete opportunity for sustainable industrial and biotechnological applications (biocatalysis, agriculture and bioremediation) and contributing to the circular bioeconomy.

## 5. Limitations of the Study

The study relies solely on classical biochemical assays and zymography. While these approaches are informative for assessing enzyme activity, they do not allow for protein identification or quantification. The integration of mass spectrometry-based proteomics would have significantly enriched the data by enabling detailed secretome profiling and the identification of specific isoenzymes. The use of only one type of cultivation substrate limits this study’s generalizability. It is well established that substrate composition strongly influences enzymatic secretion in Pleurotus species. Testing alternative substrates would provide insights into the adaptability and inducibility of the fungal secretomes. In this study, the enzymatic activity has been assessed at three distinct time points (days 23, 34, and 50) that represent key developmental stages. However, adding intermediate time points could have evidenced transient enzymatic peaks or early-stage regulation. Although the manuscript highlights the biotechnological relevance of the findings, functional application experiments such as substrate degradation and biomass hydrolysis could demonstrate the utility of the identified enzymatic profiles in real-world scenarios.

## Figures and Tables

**Table 1:** Protein concentration and total proteolytic activity of secretomes.

	Proteins (mg prot/g)			Total Proteolytic Activity (U/g)		
	Day 23	Day 34	Day 50	Day 23	Day 34	Day 50
*P. eryngii*	* 1.33 ± 0.11 ^a^	* 2.74 ± 0.16 ^b^	* 1.97 ± 0.15 ^c^	* 589.63 ± 12.85 ^a^	* 790.33 ± 9.37 ^b^	* 773.93 ± 19.34 ^b^
*P. ostreatus*	0.94 ± 0.06 ^a^	1.79 ± 0.14 ^b^	1.26 ± 0.11 ^c^	512.93 ± 12.27 ^a^	594.95 ± 13.06 ^b^	603.52 ± 8.11 ^b^

The concentrations of protein samples were determined by the Bradford method as mg proteins/g of the substrate, whereas proteolytic activities were assessed by azocasein as U/g of the substrate. Values are reported as the mean ± SD of two extracts analyzed in triplicate (n = 6). Different letters within the same row and asterisks within the same column indicate a statistically significant difference (one-way ANOVA followed by Tukey’s post hoc test, *p* < 0.05).

**Table 2:** Polysaccharide-hydrolyzing activity of secretomes.

	*P. eryngii* (U/g)			*P. ostreatus* (U/g)		
Enzymes	Day 23	Day 34	Day 50	Day 23	Day 34	Day 50
α-glucosidase	* 0.37 ± 0.01 ^a^	* 0.31 ± 0.03 ^b^	0.13 ± 0.03 ^c^	0.25 ± 0.02 ^a^	0.18 ± 0.03 ^b^	0.11 ± 0.01 ^c^
α-amylase	0.48 ± 0.02 ^a^	* 0.29 ± 0.01 ^b^	0.19 ± 0.04 ^c^	0.42 ± 0.06 ^a^	0.21 ± 0.03 ^b^	0.14 ± 0.02 ^c^
endoglucanase	0.22 ± 0.06 ^a^	* 0.51 ± 0.05 ^b^	* 0.36 ± 0.02 ^c^	0.22 ± 0.06 ^a^	0.39 ± 0.05 ^b^	0.25 ± 0.05 ^a^
β-cellobiohydrolase	* 0.51 ± 0.04 ^a^	* 0.72 ± 0.02 ^b^	* 0.39 ± 0.03 ^c^	0.33 ± 0.04 ^a^	0.51 ± 0.02 ^b^	0.27 ± 0.03 ^a^
β-glucosidase	* 0.97 ± 0.02 ^a^	* 1.15 ± 0.03 ^b^	* 0.83 ± 0.02 ^c^	0.73 ± 0.03 ^a^	0.94 ± 0.02 ^b^	0.71 ± 0.04 ^a^
β-xylanase	* 0.43 ± 0.02 ^a^	* 0.77 ± 0.03 ^b^	* 0.57 ± 0.03 ^c^	0.28 ± 0.02 ^a^	0.60 ± 0.03 ^b^	0.28 ± 0.03 ^a^
β-glucuronidase	* 0.41 ± 0.02 ^a^	0.55 ± 0.03 ^b^	* 0.45 ± 0.04 ^a^	0.31 ± 0.02 ^a^	0.49 ± 0.03 ^b^	0.22 ± 0.04 ^c^
α-arabinosidase	* 0.28 ± 0.06 ^a^	* 0.61 ± 0.03 ^b^	* 0.38 ± 0.02 ^c^	0.16 ± 0.06 ^a^	0.640 ± 0.03 ^b^	0.24 ± 0.02 ^a^
α-mannosidase	0.18 ± 0.05 ^a^	* 0.45 ± 0.02 ^b^	* 0.31 ± 0.03 ^c^	0.22 ± 0.05 ^a^	0.35 ± 0.02 ^b^	0.23 ± 0.03 ^a^

Values are reported as the mean ± SD of two extracts analyzed in triplicate (n = 6). For each *Pleurotus* species, different letters within the same row indicate a statistically significant difference. Asterisks indicate a statistically significant difference between the two species at each time point (one-way ANOVA followed by Tukey’s post hoc test, *p* < 0.05).

**Table 3:** Lipolytic activity of secretomes.

	*P. eryngii* (U/g)			*P. ostreatus* (U/g)		
Substrates	Day 23	Day 34	Day 50	Day 23	Day 34	Day 50
C2	0.01 ± 0.001 ^a^	* 0.041 ± 0.002 ^b^	* 0.047 ± 0.004 ^b^	0.008 ± 0.001 ^a^	0.014 ± 0.002 ^b^	0.019 ± 0.002 ^b^
C4	0.020 ± 0.006	* 0.030 ± 0.001 ^a^	0.060 ± 0.007 ^b^	0.016 ± 0.002 ^a^	0.022 ± 0.004 ^a^	0.053 ± 0.003 ^b^
C10	* 0.53 ± 0.02 ^a^	0.58 ± 0.02 ^b^	* 0.62 ± 0.04 ^b^	0.40 ± 0.02 ^a^	0.55 ± 0.04 ^b^	0.50 ± 0.01 ^b^
C16	* 0.29 ± 0.01 ^a^	* 0.41 ± 0.05 ^b^	* 0.44 ± 0.01 ^b^	0.24 ± 0.01 ^a^	0.32 ± 0.01 ^b^	0.30 ± 0.02 ^b^

C2: p-nitrophenyl acetate, C4: p-nitrophenyl butyrate, C10: p-nitrophenyl decanoate and C16: p-nitrophenyl palmitate. Values are reported as the mean ± SD of two extracts analyzed in triplicate (n = 6). For each Pleurotus species, different letters within the same row indicate a statistically significant difference. Asterisks indicate a statistically significant difference between the two species at each time point (one-way ANOVA followed by Tukey’s post hoc test, *p* < 0.05).

## Data Availability

Data are contained within the article.
